# The Causal Relationship Between Portal Usage and Self-Efficacious Health Information–Seeking Behaviors: Secondary Analysis of the Health Information National Trends Survey Data

**DOI:** 10.2196/17782

**Published:** 2021-01-27

**Authors:** Jaeyoung Park, Muxuan Liang, Jordan M Alpert, Richard F Brown, Xiang Zhong

**Affiliations:** 1 Department of Industrial and Systems Engineering University of Florida Gainesville, FL United States; 2 Public Health Sciences Division Fred Hutchinson Cancer Research Center Seattle, WA United States; 3 Department of Advertising University of Florida Gainesville, FL United States; 4 Department of Health Behavior and Policy Virginia Commonwealth University School of Medicine Richmond, VA United States

**Keywords:** causal inference, instrumental variable, directed acyclic graph, patient portal, self-efficacy

## Abstract

**Background:**

Patient portals have drawn much attention, as they are considered an important tool for health providers in facilitating patient engagement. However, little is known about whether the intensive use of patient portals contributes to improved management of patients’ health in terms of their confidence in acquiring health information and exercising self-care. There is a lack of randomized trials with these outcomes measured both pre- and postadoption of patient portals.

**Objective:**

The aim of this study was to examine the causal relationship between the usage of patient portals and patients’ self-efficacy toward obtaining health information and performing self-care.

**Methods:**

This study was a secondary data analysis that used data from a US national survey, the National Cancer Institute’s Health Information National Trends Survey 5 Cycle 1. Patient portal usage frequency was used to define the treatment. Survey items measuring self-efficacy on a Likert-type scale were selected as the main outcomes, including patients’ confidence in obtaining health information and performing self-care. To establish causality using survey data, we adopted the instrumental variables method. To determine the direction of the causal relationship in the presence of high-dimensional confounders, we further proposed a novel testing framework that employs conditional independence tests in a directed acyclic graph. The average causal effect was measured using the two-stage least squares regression method.

**Results:**

We showed that frequently using patient portals improves patients’ confidence in obtaining health information. The estimand of the weighted average causal effect was 0.14 (95% CI 0.06-0.23; *P*<.001). This means that when increasing the portal usage intensity, for instance, from 1-2 times to 3-5 times per year, the expected average increase in confidence level measured on a Likert-type scale would be 0.14. However, we could not conclusively determine the causal effect between patient portal usage and patients’ confidence in exercising self-care.

**Conclusions:**

The results support the use of patient portals and encourage better support and education to patients. The proposed statistical method can be used to exploit the potential of national survey data for causal inference studies.

## Introduction

Given the growing evidence showing that patient engagement improves health outcomes and reduces health care costs, patient portals have drawn much attention. A patient portal is a secure online health platform linked to a patient’s personal medical record that is 24/7 accessible from any location with an internet connection. Patient portals are considered an important tool for health providers in facilitating patient engagement [1,2]. Characteristics of portal users [3] and barriers to portal adoption [4,5] have been extensively investigated. Regarding the facilitators, it was found that patients who believed the patient portal was empowering demonstrated a higher intention to use [6]. The relationship between the internal use (quantified using engagement measurement) of online health platforms and external growth (its reach), and the social support activities related to users’ participation has been examined as well [7,8]. Research has also focused on whether portal or secure messaging usage can affect the frequency of patients’ office visits [9-15] and appointment adherence [16-19].

Nonetheless, little is known about whether patient portal usage contributes to the improved management of patients’ health in terms of health literacy, communication, confidence in acquiring health information, and the self-monitoring and self-care of health. Interviews have indicated that patient’s perception of access to online records are associated with a greater focus on their health and more proactive involvement in self-care [20,21]. However, there is a lack of randomized trials with self-efficacy outcomes measured both pre- and postportal adoption. Consequently, a causal link between patients’ portal usage and patients’ health information–seeking behaviors (self-efficacy) has not been formally established.

Despite the passage of the Health Information Technology for Economic and Clinical Health (HITECH) Act and the recommendations by the US Institute of Medicine [22], the overall adoption rate of portals remains low [23]. A better understanding of the impact of patient portals and the benefit to patients is needed to increase adoption. To this end, we used the Health Information National Trends Survey (HINTS) data [24] to examine the causal relationship between the usage of patient portals and patients’ self-efficacy in obtaining health information and performing self-care.

## Methods

### Study Setting

This study was a secondary data analysis that used data from the National Cancer Institute’s HINTS 5 Cycle 1. HINTS 5 Cycle 1 is a cross-sectional survey of a nationally representative sample of US adults used to assess the impact of the health information environment. The survey was conducted from January 2017 through May 2017 using a self-administered mail questionnaire. Out of 10,265 surveys sent out, data were collected from 3285 (32% response rate) respondents [24]. The characteristics of these respondents are summarized in Table S1 in Multimedia Appendix 1.

Patients’ self-efficacy in obtaining health information and performing self-care were considered as the outcomes. Survey items measuring patient self-efficacy were selected based on the Institute of Medicine’s recommendations for promotion of patient portals to increase quality of care and reduce medical errors [22], as well as claims that health information technology, like portals, could increase patients’ self-efficacy for managing their conditions [25-28]. Perceived confidence was assessed with the following questionnaire items: (1) “Overall, how confident are you that you could get advice or information about health or medical topics if you needed it*?*” (ConfidentGetHealthInfo, Y_1_); (2) “Overall, how confident are you about your ability to take good care of your health? (OwnAbilityTakeCareHealth, Y_2_).”

These measures were captured on a 5-point Likert-type scale where a higher score indicated greater confidence. Variables on patients’ age, gender, race, ethnicity, marital status, education, employment status, household income, and insurance status were considered to be confounders in the study. Information on patients’ portal activities was elicited by the following questionnaire item: “How many times did you access your online medical record in the last 12 months? (0/1 to 2 times/3 to 5 times/6 to 9 times/10 or more times).”

Different usage frequencies were considered to be different levels of treatment. For the instrumental variable (IV), we used the following questionnaire item: “Have any of your health care providers including doctors, nurses, or office staff ever encouraged you to use an online medical record? (yes/no).”

Missing values were sparse and were handled in several ways depending on the variable type: samples missing the outcomes were discarded; for the IVs, missing responses were replaced by “no encouragement”; and for the confounders, missing responses were imputed by the Multivariate Imputation by Chained Equations (MICE) [29] method with the IV and the confounders used as input variables for that method.

### Establishing a Causal Relationship

We aimed to test the hypothesis that the exposure to patient portals, or intensively using a patient portal, will improve patients’ self-efficacy outcomes. However, since we only used a one-time outcome measurement for each individual, we could not construct the before-after treatment contrast to directly measure the treatment effect. Further, without a randomized experimental design, the causality can be obscured by confounders. To address these issues, we adopted the IV method for causal inference in observational studies [30]. In these studies, IVs are used to adjust for both observed and unobserved confounding effects and to help identify a contrast of outcomes in the absence of temporal data.

We identified the following item as the binary variable and used it as the IV: “Have any of your health care providers including doctors, nurses, or office staff ever encouraged you to use an online medical record?”

Encouragement plays an influential role on patients’ use of portals, as care providers’ endorsement is an important factor in the adoption of these tools [6-8,31]. However, provider referrals to use patient portals have long been known to vary by race and socioeconomic status and are thought to be dependent on whether the provider believes that the patient will use the portal [32]. The same is true for the HINTS data we used, where income and education were observed to be associated with recommendation, patient portal usage, and self-efficacy. Thus, the requirement of no confounding between the IV and the outcome might not have been met if encouragement (or other candidate IVs, such as internet savviness) were used as an IV.

To further determine whether patient portal usage causes an improvement in self-efficacy, or vice versa, we proposed a testing framework that could both address the confounding issue and determine the direction of the causal relationship. Our testing framework was based on causal directed acyclic graphs (DAGs), which are used as a graphical tool to visually represent and understand the concepts of exposure, outcome, causation, and confounding [33]. To identify an appropriate IV, we generalized the criteria of IVs to allow for known (or observable) confounding among the IV, treatment, and outcome; and unknown (or unobservable) confounding between the treatment and outcome [30]. With this separation of confounders, only the known common confounders were essential for examining the causal relationship. To further identify the direction of causality, a testing framework employing conditional independence tests [34,35] was developed. The detailed description of the testing framework can be found in Multimedia Appendix 2.

### Measuring Causal Effect

The proposed DAG-based testing framework aimed to qualitatively evaluate the causality. To quantify the treatment effect, two-stage least squares (TSLS) regression models were built. As the treatment (portal usage) has multiple levels (eg, 1-2 times and 3-5 times annually are different levels), the traditional average treatment effect is not identifiable. However, Angrist and Imbens [36] showed that an estimand, essentially a weighted average of per-unit average causal effects, is identifiable and can be estimated by TSLS regression models. The two major assumptions therein, independence and monotonicity of ordinal treatment effect, were thus naturally satisfied in our causal framework.

## Results

### Portal Enrollment

After removing the samples with too many missing data, we identified 3198 participants among the 3285 survey respondents. Among these 3198 participants, 1003 (31%) were self-reported patient portal users. For demographic and socioeconomic variables, the user group and the nonuser group had different characteristics (see Table S1 in Multimedia Appendix 1). Patients younger than 65 years old and females were more likely to be patient portal users. Moreover, participants who self-reported as White, married, and non-Hispanic were more likely to be users. In terms of income, higher income was positively correlated with a greater likelihood of being a portal user. Likewise, participants who were employed were more likely to use portals than those who were retired or unemployed. Compared to nonusers, portal users had a higher level of education (eg, undergraduate or postgraduate). Finally, compared to those covered by employer-provided insurance, patients who were covered by private insurance, Medicare, or Medicaid were less likely to enroll in patient portals.

### Encouragement and Portal Usage

Next, we characterized the users’ portal usage behavior. Of the 1003 portal users, 49% (496) reported using portals 1-2 times in the past 12 months, 31% (313) reported using them 3-5 times, 10% (104) reported using them 6-9 times, and 9% (90) reported using them more than 9 times.

Of the 3198 participants, 1375 (43%) were encouraged to use patient portals and 1823 (57%) were not. Among the 1375 respondents who were encouraged, 549 (40%), 383 (28%), 267 (19%), and 176 (13%) individuals never used a portal, used a portal 1-2 times, 3-5 times, and more than 5 times, respectively. In contrast, there were 1646 (90%), 113 (6%), 46 (3%), and 18 (1%) participants in the nonencouraged group, respectively. It was evident that the IV and the treatment were significantly associated, which was verified by a chi-square test (*P*<.001).

### Encouragement and Self-Efficacy

There were more patients with positive responses for the self-efficacy outcomes in the patient population who were recommended to use portals. The distributions of each outcome variable conditioning on the value of the IV are displayed in Table 1, and chi-square test results are provided. Among the 3198 participants, 3111 and 3165 participants were identified as those who answered questions Y1 and Y2, respectively. For ConfidentGetHealthInfo, individuals in the encouraged group (G_e_) were more likely to be completely confident or very confident than those in the nonencouraged group (G_n_; G_e_: 907/1353, 67%; G_n_: 1000/1758, 57%; *P*<.001). For OwnAbilityTakeCareHealth*,* G_e_ patients were slightly more confident in self-care (G_e_: 998/1364, 73%; G_n_: 1222/1801, 68%; *P*=.001). All the comparisons suggest that the IV and the outcomes were significantly associated.

**Table 1:** Chi-square test results for the association between encouragement to use patient portals and self-efficacy outcomes.

Outcomes		IV^a^: encouragement to use patient portals		*P* value
		Yes, n (%)	No, n (%)	
**ConfidentGetHealthInfo, Y_1_**		1353 (100)	1758 (100)	<.001
	Not confident at all	21 (1.5)	50 (2.8)	
	A little confident	56 (4.1)	108 (6.1)	
	Somewhat confident	369 (27.3)	600 (34.1)	
	Very confident	566 (41.8)	607 (34.5)	
	Completely confident	341 (25.2)	393 (22.4)	
**OwnAbilityTakeCareHealth, Y_2_**		1364 (100)	1801 (100)	.001
	Not confident at all	15 (1.1)	31 (1.7)	
	A little confident	31 (2.3)	79 (4.4)	
	Somewhat confident	320 (23.5)	469 (26.0)	
	Very confident	652 (47.8)	815 (45.3)	
	Completely confident	346 (25.4)	407 (22.6)	

^a^IV: instrumental variable.

### Causal Relationship Between Patient Portal Usage and Self-Efficacy

Following the testing procedure described in Multimedia Appendix 2, conditional independence tests were conducted. Test A tested the hypothesis that, given the common confounders and the treatment, the IV is conditionally independent with the outcome. This test examined whether higher self-efficacy is a cause for increased portal usage. Test B tested the hypothesis that the IV and the outcome are conditionally independent, given the common confounders alone. This test examined whether the increase in portal usage is a cause for higher self-efficacy. A *P* value <.05 was used as the statistical significance level for the overall test. The results are shown in Table 2.

**Table 2:** Results of conditional independence tests.

Outcomes	*P* value	
	Test A: Z ⊥ Y ∣ 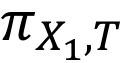	Test B: Z ⊥ Y ∣ 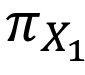
ConfidentGetHealthInfo	.28	.02
OwnAbilityTakeCareHealth	.42	.23

**Figure 1:**
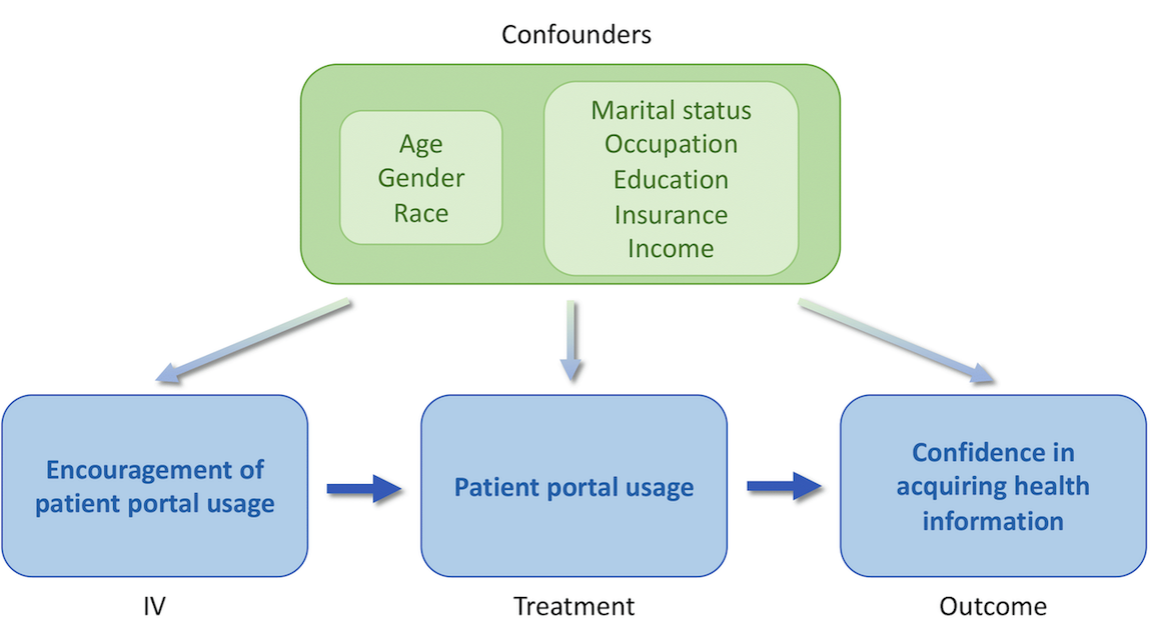
The causal relationship among encouragement, portal usage, and confidence in seeking health information with the confounders. IV: instrumental variable.

For ConfidentGetHealthInfo, we could not reject the hypothesis for Test A (overall *P*=.28) but could reject Test B (overall *P*=.02), meaning that portal usage did have a causal effect on self-efficacy toward obtaining health information. This relationship is illustrated in Figure 1. However, for OwnAbilityTakeCareHealth, we could not reject the hypothesis for either Test A (overall *P*=.42) or Test B (overall *P*=.23). In this case, we could not determine the causal relationship between patients' portal usage and patients’ confidence in exercising self-care.

### Treatment Effect of Patient Portal Usage on Self-Efficacy

A TSLS regression model was built to quantify the treatment effect of portal usage on self-efficacy toward acquiring health information. The estimand of the weighted average causal effect was 0.14 (95% CI 0.06-0.23; *P*<.001). This indicates that, when increasing the portal usage intensity, for instance, from 1-2 times to 3-5 times per year, the expected average increase in the reported confidence level (measured with a Likert-type scale) would be 0.14. It can be concluded that the more intensely patients engage with portals, the more confident they are in obtaining health information.

## Discussion

### Identifying Causality

Existing work on survey analysis mainly focuses on the strength of the association between questionnaire items and targeted outcomes [37,38], as there is a lack of understanding regarding the causal path diagram and a lack of methods for determining if the path diagram is congruent with the data. In this study, we developed a new framework that allowed us to detect causality and estimate the magnitude of the treatment effect represented in the path diagram using the HINTS 5 survey. Our framework was able to eliminate the estimation bias due to unmeasured confounders, and guarantee the test efficiency given the limited sample size of the survey. Moreover, the testing framework was robust to the choice of IVs. In addition to using “encouragement” as an IV, we tested internet savviness as an alternative IV and found the conclusion was consistent (see Multimedia Appendix 2).

### Effect of Portal Usage

Although we should be cautious in interpreting the relationship between using patient portals and self-efficacy as causal, we did observe a co-occurrence of better outcomes and increased patient portal usage intensity. Despite the benefits of patient portals being well documented [39,40], rates of usage remain low [41], and increasing the adoption of patient portals would provide more patients a trusted source of personalized information. Because patients can be easily misinformed by the large amount of inaccurate information online [42], using patient portals allows patients and their caregivers to stay connected with their providers. This occurs through easy access to their own health information and the ability to contact providers via secure messaging if they have questions or concerns. Our framework further confirmed that portal usage does positively affect confidence in obtaining health information. However, we did not observe a similar level of significance to conclude that higher portal usage will lead to higher self-efficacy in performing self-care. It is possible that other confounding factors, such as income and education levels [43], might have had a more powerful effect on the outcome.

### Encouraging Patients’ Portal Use

Our analysis shows that being encouraged to use patient portals positively affects the intensity of portal usage, which in turn influences people’s confidence in acquiring health information. Other studies have reported that actively using patient portals, such as sending messages to physicians and viewing prescription and lab results, is positively associated with high-quality physician-patient relationships and patients’ confidence to understand health information. It is evident that a trusting physician-patient relationship helps promote healing and remove barriers to obtaining medical information [44]. However, efforts to increase the adoption of patient portals often rely on pamphlets and flyers [45] or hurried conversations by medical staff. Given the potential benefits of patient portals and our findings that encouragement affects usage, we believe that an innovative intervention is necessary to increase patient adoption and usage, which ultimately can lead to significant improvements in patient outcomes.

### Limitations

The study was conducted using HINTS 5 Cycle 1 data, which were collected in 2017. The study can be improved by combining multiple data sets, including ones published recently [46]. The power of the test can be increased by including more samples, which can potentially help identify the causal relationship between portal usage and self-care. It is worth noting that survey designs are not identical across all three cycles of HINTS 5. For instance, the outcome of ConfidenceGetHealthInfo is available in Cycle 1 (2017) and Cycle 3 (2019) but not in Cycle 2 (2018). Considerable effort will be needed to merge these data sets. Furthermore, the HINTS database contains rich information beyond the variables that were used for this study, and other aspects (eg, health disparities) related to both portal usage and self-efficacy behaviors should be explored.

The establishment of the causal relationship between portal usage and patients’ self-efficacy demands that encouragement to use portals is not based on patients’ self-efficacy. We have observed that many health care organizations have integrated the facilitation of portal enrollment into their new patients’ registration protocol [47]. In addition, portal use encouragement also occurs during a patient’s interaction with the front desk when addressing billing and appointment scheduling, as patient portals can be an alternative venue to handle these services. These scenarios correspond to the cases that nurses or office staff encourage patients to use an online medical record. These situations account for the majority of the encouragement during patient encounters and thus support the selection of encouragement as an IV. However, as for personal encouragement from a physician, we are unaware of any health care organizations that have policies or incentives for providers to systematically encourage all patients to use portals, meaning that encouragement can be at the providers’ discretion [48]. Therefore, collecting physicians’ input concerning their patterns of encouragement would be valuable for further justifying the validity of using encouragement as an IV. To ensure the rigor of our results, we further tested internet savviness as an alternative IV and found a consistent conclusion (see Multimedia Appendix 2).

Although the confounders between the treatment and the outcome can be completely unmeasured, we still require the common confounders to be fully observable. The identification of common confounders largely relies on domain knowledge. It is worth noting that the choice does not have to be unique. In our casual diagram, adding variables to the common confounder set still results in a valid choice. However, chi-square tests can be sensitive to the choice of confounders when the sample size is not sufficiently large [49]. Therefore, to make the results more robust, more survey respondents are needed.

### Conclusions

Since the Affordable Care Act mandated portal usage, enthusiasm for portals has declined; however, this study found that using patient portals improves patients’ confidence in obtaining advice or information about health or medical topics. Our findings thus attest to the benefit of patient portals and to providing better support and education to patients. In addition, our proposed statistical method exploits the potential of using national survey data such as the HINTS program to examine causal effects to obtain new insights. Theoretically, the treatment effect can be heterogeneous based on different patient characteristics. There is thus a need to develop a testing framework that can identify the disparity in causal effects. For justifying the clinical insights identified in this study, we cannot solely rely on patients’ self-reported outcomes, but should also survey physicians on their perception of patients’ self-efficacy. Furthermore, how physicians make encouragement decisions should also be investigated, and a randomized controlled study with pre- and posttreatment outcomes being clearly documented is necessary to fully understand the treatment effect on self-efficacy outcomes.

## Appendix

Multimedia Appendix 1 Table S1.

Multimedia Appendix 2 Methods.

## Abbreviations

DAG: directed acyclic graph
HINTS: Health Information National Trends Survey
HITECH: Health Information Technology for Economic and Clinical Health
IV: instrumental variable
MICE: Multivariate Imputation by Chained Equations
TSLS: two-stage least squares
