# Supplementary material for: The Causal Relationship Between Portal Usage and Self-Efficacious Health Information–Seeking Behaviors: Secondary Analysis of the Health Information National Trends Survey Data
Source: J Med Internet Res. 2021 Jan 27;23(1):e17782. doi: 10.2196/17782 (PMC7875689; doi:10.2196/17782)
Supplement: Multimedia Appendix 1 [file jmir_v23i1e17782_app1.pdf]

Table S1. Characteristic of respondents in the non-user group and the user group

		Non-user		User		*P* value
Characteristic		n=2195	(%)	n=1003	(%)	
Age						<0.001
	18 - 30	155	7.1	74	7.2	
	31 - 45	383	17.4	226	19.0	
	46 - 64	844	38.5	432	39.9	
	65+	813	37.0	271	33.9	
Gender						0.006
	Female	1261	57.4	629	59.1	
	Male	934	42.6	374	40.9	
Race						<0.001
	White	1548	70.5	778	72.7	
	Black or African American	400	18.2	115	16.1	
	Asian	96	4.4	58	4.8	
	Other	151	6.9	52	6.3	
Hispanic						<0.001
	Hispanic	341	15.5	92	13.5	
	Not Hispanic	1854	84.5	911	86.5	
Marital status						<0.001
	Married	1099	50.1	664	55.1	
	Divorced or Separated	427	19.5	159	18.3	
	Widowed	284	12.9	53	10.5	
	Single	385	17.5	127	16.0	
Education						<0.001
	<= 12 yrs	696	31.7	135	26.0	
	College	672	30.6	275	29.6	
	College graduate	509	23.2	325	26.1	
	Postgraduate	318	14.5	268	18.3	
Occupation status						<0.001
	Employed	1022	46.6	607	50.9	
	Unemployed	273	12.4	71	10.8	
	Retired	733	33.4	281	31.7	
	Disabled	167	7.6	44	6.6	
Income						<0.001
	$0 to $19,999	514	23.4	78	18.5	
	$20,000 to $49,999	668	30.4	203	27.2	
	$50,000 to $99,999	628	28.6	352	30.6	
	$100,000 or more	385	17.5	370	23.6	
Insurance - Employ coverage						<0.001
	No	1164	53.0	309	46.1	
	Yes	1031	47.0	694	53.9	
Insurance - Private						0.008
	No	1805	82.2	863	83.4	
	Yes	390	17.8	140	16.6	
Insurance - Medicare						<0.001
	No	1335	60.8	724	64.4	
	Yes	860	39.2	279	35.6	
Insurance - Medicaid						<0.001
	No	1770	80.6	922	84.2	
	Yes	425	19.4	81	15.8	
