# Supplementary material for: The Causal Relationship Between Portal Usage and Self-Efficacious Health Information–Seeking Behaviors: Secondary Analysis of the Health Information National Trends Survey Data
Source: J Med Internet Res. 2021 Jan 27;23(1):e17782. doi: 10.2196/17782 (PMC7875689; doi:10.2196/17782)
Supplement: Multimedia Appendix 2 [file jmir_v23i1e17782_app2.docx]

# **Multimedia Appendix 2**

We present in this supplementary document a detailed description of the methods developed to identify causal relationships using survey (non-temporal) data.

# **Causal diagram**

Our testing framework is based on the causal directed acyclic graphs (DAGs), which are used as a graphical tool to visually represent and understand the concepts of exposure, outcome, causation, and confounding [33]. In a DAG, causal relationships are represented by arrows between variables, pointing from cause to effect. We consider a DAG with only one edge the direction of which is not determined, see Figure A1. In this setting, X is identified as the confounder as it points to both T and Y. Z has a causal effect on T. However, the relationship between T and Y is not clear. If it is the case that the arrow points from T to Y, then, this DAG represents the classical causal diagram with T as the treatment, Y as the outcome, and Z as the instrumental variable (IV). Given that there exists an arrow between T and Y, our overall goal is to identify the direction of the arrow, which determines the exact causal relationship between T and Y.


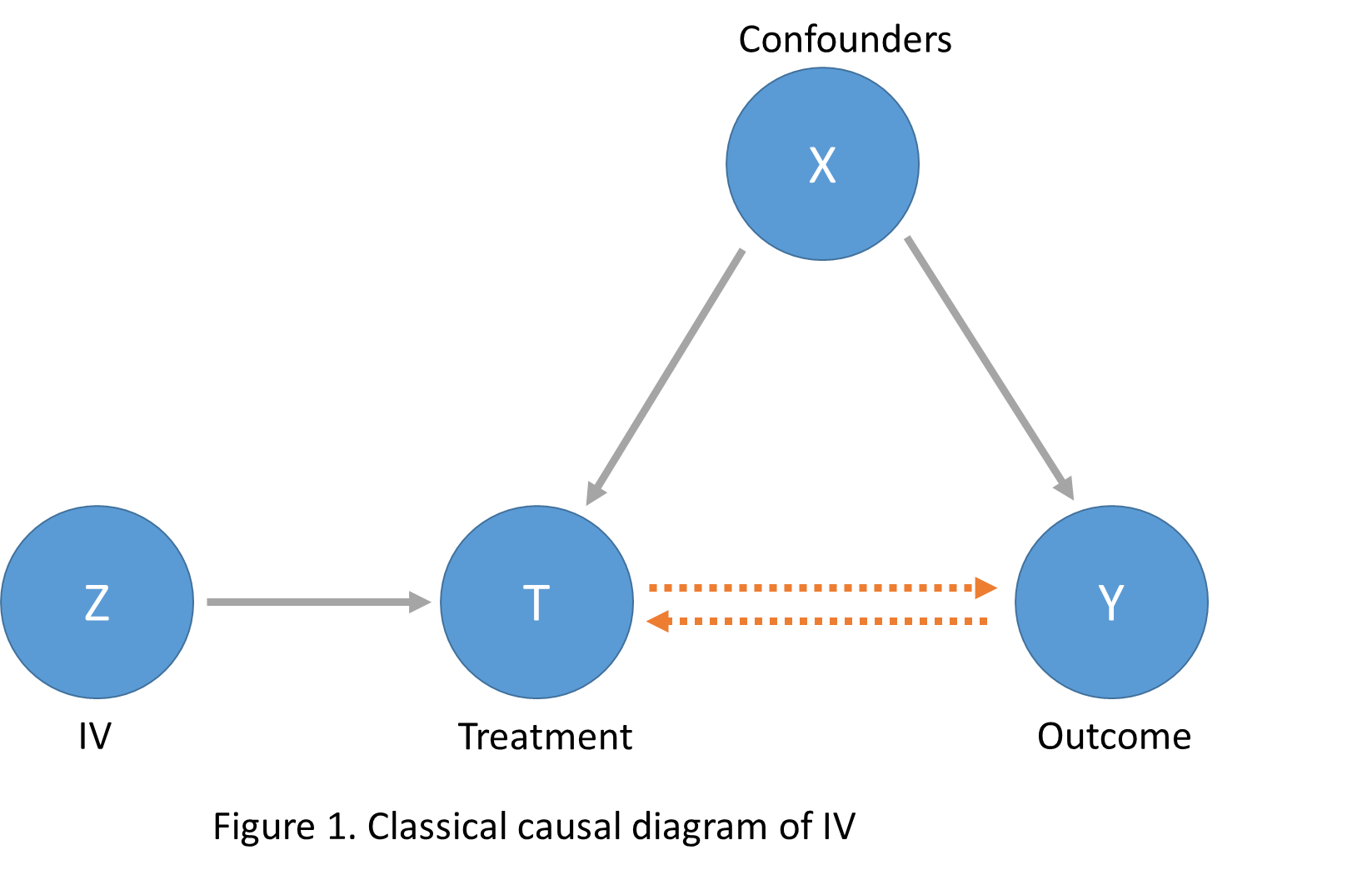


**Figure A1**. A classical causal diagram with Z as an IV.

# **Instrumental variables method for causal inference**

With survey data, one cannot obtain the contrast of the outcomes before and after the intervention to directly measure the treatment effect. Therefore, IVs are introduced to relate Y and T by constructing other forms of contrast of outcomes. An IV (Z) should have a causal effect on the treatment (T), and affect the outcome (Y) only through T. This means that Z does not have a direct influence on Y, neither does Y influence Z, which is referred to as the exclusion restriction. In addition, there should not be confounding for the effect of Z on Y [30]. If there exists a variable Z satisfying these conditions, it can be used as an IV. Then, one can construct a contrast of Y based on the value of Z. If T does affect Y, then, there is a link from Z through T to Y. The distribution of Y conditioning on different levels of Z should differ. In this way, even without temporality in data, the casual effect can still be established.

However, it might not be easy to identify a variable Z that possesses the ideal property of IVs. The condition of no confounding between Z and Y cannot be easily justified in observational studies, and the judgment highly depends on the domain knowledge. A more realistic assumption is to allow for some known part of X to contribute to the confoundedness between Z, T, and Y. Therefore, in our causal analysis, we further generalize the criteria of IVs to allow for known (or observable) confounding among Z, T, and Y and unknown (or unobservable) confounding between T and Y. Consequently, we split confounders X into two disjoint groups, $X_{1}$ and $X_{2}$, as illustrated in Figure A2. Variables in $X_{1}$ are the known common confounders between Z, T, and Y. Variables in $X_{2}$ are the confounders between T and Y, and $X_{2}$ is not required to be fully measurable. If Z is independent with both Y and X, then $X_{1}$ is an empty set, and the causal diagram degenerates to the classical IV framework as shown in Figure A1.


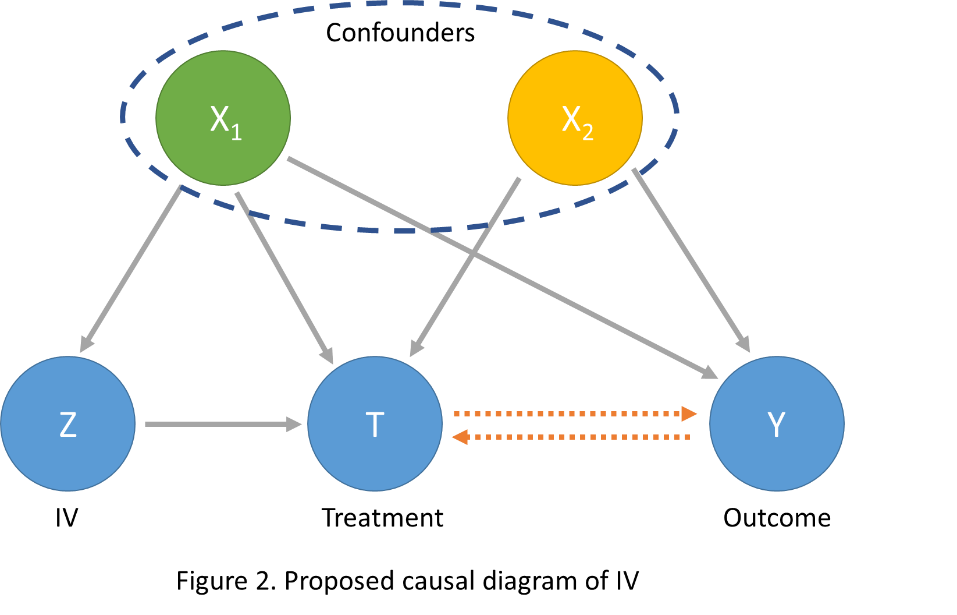


**Figure A2**. A generalized causal diagram with known and unknown confounders.

# **Conditional independent tests for high-dimensional confounders**

With the DAG described in Figure A2, we employ the following framework motivated by the d-separation concept [33] to determine causality as tests of conditional independence. To be more specific, we aim to test whether T causes Y, or is caused by Y, which is not specified in Figure 2A. If and only if T causes Y, then, Z and Y are not conditionally independent given $X_{1}$. Further, Z and Y should be conditionally independent given both T and $X_{1}$. This is valid regardless of the existence of $X_{2}$. On the other hand, if T is caused by Y, the opposite is true: Z and Y are conditionally independent given $X_{1}$, and Z and Y are not conditionally independent given T and $X_{1}$. Thus, once $X_{1} \subseteq X$ is identified, tests of conditional independence can be conducted to determine the direction of the arrow.

In a typical setting, Z is binary, Y is binary or ordinal, but $X_{1}$ can be a high-dimensional vector that contains variables in different formats (e.g., continuous and categorical). Therefore, instead of directly testing $Z\perp Y|X_{1}$, we propose a more efficient testing method based on Theorem A1.

**Theorem A1**. Given $Z\perp Y|U$, and define $\pi_{U}:=P(Z=1|U)$, then, $Z \perp Y \left| U\to Z \perp Y \right|\pi_{U}$.

We introduce $\pi_{X_{1}}=P\left( Z=1 | X_{1} \right),$the probability that the IV takes value 1 given confounders $X_{1}$.This probability can be obtained using a logistic regression model with a lasso penalty when $X_{1}$is high-dimensional [35]. Then, if we can prove that given $\pi_{X_{1}}$, Z and Y are conditionally dependent, then, we can claim that given $X_{1}$, Z and Y are conditionally dependent. Since $\pi_{X_{1}}\in$ [0,1], we further discretize it into different levels based on its quartiles (denoted as $G_{1}$, $G_{2}$, $G_{3}$, and $G_{4}$), and denoted this discrete variable as $\bar{\pi}_{X_{1}}$. Since the test of $Z\perp Y |\bar{\pi}_{X_{1}}$ only concerns discrete variables, it can be done using Chi-square tests [34]. The null hypothesis is that Y and Z are independent conditioning on $\bar{\pi}_{X_{1}}$, for all levels of $\bar{\pi}_{X_{1}}$, and the alternative hypothesis is that for at least one level of $\bar{\pi}_{X_{1}}$, Y and Z are not conditionally independent. If we reject $Z\perp Y|\pi_{X_{1}}$, then, there is a causal effect of T on Y.

Notably, if $Z\perp Y |\bar{\pi}_{X_{1}}$, we still cannot conclude it is Y that causes T. We need to check if Z and Y are not conditionally independent given T and $X_{1}$. This can be checked by replacing U by ${(T,X}_{1})$in Theorem A1, and calculating the conditional probability $\pi_{X_{1},T}=P(Z=1|X_{1},T)$. If given $\pi_{X_{1},T}$, Z and Y are not conditionally independent, then, one can imply that given $X_{1}$ and T, Z and Y are not conditionally independent. If we reject $Z\perp Y|\pi_{X_{1},T}$, then, there is a causal effect of Y on T.

The proposed method has the following advantages. First, it is applicable to observational data and data lack of temporal order such as survey data. In the latter case, without knowing which event happens first, the direction of the causal path is unclear. Our method is able to detect the direction and establish causality when an appropriate IV can be identified. Second, the choice of IV is broadened in our analysis comparing to the classical IV framework. With the existence of measurable common confounders (i.e., confounders related to the IV, the treatment variable, and the outcomes simultaneously), the confounders between the treatment and the outcome alone do not have to be explicitly specified or measured. Third, the common confounders can be high-dimensional. For example, hundreds of important confounders could present in surveys that contain many multiple-choice questions to characterize respondents. However, the number of respondents can be small compared to the number of variables. Here we address this issue by using a unified score and proved that it is a necessary condition for the conditional independence of the IV and the outcome given the common confounders. Using this score as a surrogate to the high-dimensional confounder, we can enable dimension reduction and boost the test efficiency significantly.

# **Proof of Theorem A1:**

First, we show that $Z \perp U | \pi_{U}$. This is equivalent to showing that for any function $h$ and $g$, we have $E[h\left( Z \right)g\left( U \right)|\pi_{U}]=E\left[ h\left( Z \right) | \pi_{U} \right]E\left[ g\left( U \right) | \pi_{U} \right].$

Direct calculation yields

$$E\left[ h\left( Z \right)g\left( U \right) | \pi_{U} \right]=E\left[ E\left[ h\left( Z \right)g\left( U \right) | U \right] | \pi_{U} \right]=E\left[ E\left[ h\left( Z \right) | U \right]g\left( U \right) | \pi_{U} \right].$$

Notice that $E\left[ h\left( Z \right) | U \right]=h\left( 1 \right)\pi_{U}+h\left( 0 \right)\left( 1-\pi_{U} \right),$

$$E\left[ E\left[ h\left( Z \right) | U \right]g\left( U \right) | \pi_{U} \right]=\left[ h\left( 1 \right)\pi_{U}+h\left( 0 \right)\left( 1-\pi_{U} \right) \right]E\left[ g\left( U \right) | \pi_{U} \right].$$

Thus, $E[h\left( Z \right)g\left( U \right)|\pi_{U}]=E\left[ h\left( Z \right) | \pi_{U} \right]E\left[ g\left( U \right) | \pi_{U} \right]$.

Now, given $Z \perp Y | U$, we want to show that $Z \perp Y | \pi_{U}$. Similarly, for any function $h$ and $g$, we have

$$E\left[ h\left( Z \right)g\left( Y \right) | \pi_{U} \right]=E\left[ E\left[ h\left( Z \right)g\left( Y \right) | U \right] | \pi_{U} \right].$$

Because $Z \perp Y |U$, we know that $E\left[ h\left( Z \right)g\left( Y \right) | U \right]=E\left[ h\left( Z \right) | U \right]E\left[ g\left( Y \right) | U \right]$.

Again, notice that $E\left[ h\left( Z \right) | U \right]=h\left( 1 \right)\pi_{U}+h\left( 0 \right)\left( 1-\pi_{U} \right)$, then, we have

$$E\left[ E\left[ h\left( Z \right)g\left( Y \right) | U \right] | \pi_{U} \right]=\left[ h\left( 1 \right)\pi_{U}+h\left( 0 \right)\left( 1-\pi_{U} \right) \right]E\left[ g\left( Y \right) | \pi_{U} \right].$$

Thus, $E\left[ h\left( Z \right)g\left( Y \right) | \pi_{U} \right]= E\left[ h\left( Z \right) | \pi_{U} \right]E\left[ g\left( Y \right) | \pi_{U} \right].$That is $Z \perp Y | \pi_{U}$.

# **Robustness of the IV method**

To examine the robustness of the testing framework with respect to the choice of IVs, we identified another variable in the HINTS data, the questionnaire item “How often do you access the Internet though each of the following?” If a participant responded “Daily” at least once to the following items, including “computer at home, “computer at work,” “computer at school,” “computer in a public place,” “on a mobile device,” and “on a gaming device,” we regarded this participant as internet savvy. Using this variable as an IV, we repeated the testing procedure and the results are shown in Table A1. The same conclusion was made: more portal usage makes patients to be more confident in getting health information. Similarly, no conclusion can be drawn regarding confidence in exercising self-care. It suggests that our conclusion is robust to the IV used in the testing framework.

**Table** **A1.** Results of conditional independence tests with internet savviness as the IV.

| Outcomes | *P* value | |
| --- | --- | --- |
|  | Test A: $Z\perp Y\vert\pi_{X_{1},T}$ | $Test B:Z\perp Y\vert\pi_{X_{1}}$ |
| ConfidentGetHealthInfo | .261 | .024 |
| OwnAbilityTakeCareHealth | .833 | .888 |
